# Effects of Mango Peel Addition on the State Diagram and Quality Changes of Ultra-Fine Mango Powder During Storage

**DOI:** 10.3390/foods15213948

**Published:** 2026-11-03

**Authors:** Min Gan, Jialin Xu, Jiuhua Zhang, Qingfu Wang

**Affiliations:** 1 Guangdong Engineering Research & Development Center for Comprehensive Utilization of Plant Fiber, Institute of Biological and Medical Engineering, Guangdong Academy of Sciences, Guangzhou 510316, China; gminwell@163.com (M.G.);; 2 School of Food Science and Engineering, Central South University of Forestry and Technology, Changsha 410004, China

**Keywords:** mango peel, ultra-fine mango powder, storage, quality characters, kinetics

## Abstract

Mango (*Mangifera indica* L. cv. Tainong) peel waste is a promising source of physicochemical and nutritional ingredients for food applications. This study aimed to explore how peel addition affects the storage stability and quality deterioration of ultra-fine mango powders, with the peel powder incorporated into mango-flesh powder at mass fractions of 8%, 16% and 24%. The state diagrams indicated that peel addition significantly lowered the water activity (*A_w_*) and raised the glass transition temperature (*T_g_*) of the powders, improving stability. Physicochemical characters including color, moisture content, *A_w_*, bulk density, angle of repose, reducing sugar, ascorbic acid, β-carotene, total phenols, flavonoids, and antioxidant activity of ultra-fine mango powders incorporated with peel were monitored and modeled during 90 days of storage at 4 °C. The results indicated that peel addition adjusted the initial color and narrowed the color changes of the stored samples, as well as inhibiting moisture adsorption and Aw. The degradation of reducing sugars and β-carotene in ultra-fine mango powders with different peel content followed first-order kinetics, whereas ascorbic acid, total phenol content (TPC), and total flavonoid content (TFC) conformed to second-order models. Peel enrichment dose-dependently increased initial bioactive levels, reduced degradation rates, and extended half-lives (*t*_1/2_). After 90 days, TPC and TFC losses dropped from 43.60% and 51.24% (pure powder) to 28.91% and 41.58% with 24% peel addition. Antioxidant activities were improved by peel addition and exhibited a strong positive correlation with TPC and TFC (R^2^ > 0.94). These results support mango peel as a sustainable functional ingredient for clean-label products and circular economy strategies.

## 1. Introduction

Mango (*Mangifera indica* L.), one of the most commercially valuable tropical fruits and rich in bioactive compounds such as ascorbic acid, polyphenols, and carotenoids, is prone to mechanical damage and microbial contamination, leading to spoilage after harvest. Mango powder, as a dominant processing product of fresh mangoes, effectively alleviates the limitations of short shelf life and seasonal supply, and is widely utilized in solid beverages, baked goods, baby foods, and functional food formulations [1]. Nevertheless, mango powder exhibits high sugar content and strong hygroscopicity, which increases its susceptibility to caking and loss of powder flowability. Additionally, its bioactive compounds (e.g., polyphenols, flavonoids, and carotenoids) degrade rapidly under improper storage conditions, compromising its nutritional quality [2]. It has been reported that ascorbic acid and total polyphenol of mango powder stored at room temperature for 90 days decreased by 65.60% and 51.79%, respectively [3]. Therefore, developing feasible strategies to enhance the storage stability of mango powder and clarify its quality deterioration mechanisms is essential for promoting the sustainable development of the mango processing industry.

During conventional mango pulp processing, mango peel is stripped from the fruit after peeling and becomes an abundant by-product, which accounts for approximately 15~20% of the total fresh fruit weight [4]. However, a significant portion of this by-product is discarded indiscriminately, leading to considerable resource waste and environmental burden. It has been well documented that mango peels have high amounts of fiber, ascorbic acid, tocopherols, phenolic compounds and carotenoids [5,6,7,8], exhibiting excellent potential to be reused as a source of functional ingredients and natural preservatives [9,10,11,12]. This suggests that incorporating mango peel powder into mango powder could serve as an alternative solution. This approach not only enhances the nutritional quality of the mango powder but also modulates its physicochemical properties and storage stability, owing to the highly nutritious components and fiber content present in mango peel powder.

In our previous research, it was found that the addition of 8~24% mango peel powder into mango flesh powder increased the total phenol concentration by 3.29~3.94-fold, total flavones by 1.52~2.24-fold, ascorbic acid by 1.08~1.29-fold, and ABTS antioxidant activities by 2.63~3.02-fold; meanwhile, the water activity, bulk density, angle of repose, and water absorption capacity decreased [13]. Fong-in et al. [14] reported that the addition of 5~10% mango peel powder to freeze-dried mango powder increased the total phenolic content and total flavonoid content, as well as improving the glass transition temperature (T_g_) from 16.03 °C (0% mango peel powder addition) to 26.62 °C (5%) and 30.07 °C (5%). Furthermore, numerous studies have demonstrated that incorporating mango peel as functional ingredient into baked products can dramatically enhance their sensory and nutritional attributes [15,16,17]. Nevertheless, these previous investigations have only evaluated the fresh-state properties of peel-supplemented mango powders, or the quality of ready-to-eat products fortified with mango peel powder. Limited literature is available regarding the long-term storage stability, moisture sorption behavior, and progressive quality degradation of peel-supplemented mango powders. To fill this research gap, the present work addresses storage-induced changes of ultra-fine mango powder incorporated with mango peel powder.

In this study, the adsorption isotherms and T_g_ of mango powder with varying additions of mango peel (8%, 16%, and 24%) were determined and fitted, and the state diagrams of these samples were established to predict the optimal storage condition. Subsequently, the effects of different levels of mango peel incorporation on the quality changes of mango powder during a 90-day storage period were systematically investigated, and kinetic models were employed to elucidate the deterioration patterns of nutritional compounds. These findings could offer technical support for the development of highly stable mango powder materials and propose a novel approach to enhance the utilization efficiency of by-products such as fruit and vegetable peels.

## 2. Materials and Methods

### 2.1. Materials

Fresh ripe mangoes (*Mangifera indica* L. cv. Tainong) with uniform maturity were purchased from a local farmers’ market in Guangzhou, China, each weighing approximately 60–80 g. All purchased mangoes were transported to the laboratory at ambient temperature (27–33 °C) then stored at 12 ± 1 °C with relative humidity 85~95% for less than 5 days before usage.

### 2.2. Preparation of Ultra-Fine Mango Powders with Various Mango Peel Powder Addition

As mango peel, the fruit’s outermost layer, is exposed to the environment and postharvest handling with potential bacterial, yeast and mold contamination, here, before processing, mangoes without decay or injuries were selected and carefully washed with tap water. According to Xu et al. [13], selected mangoes were manually peeled and pitted, and the obtained flesh was cut into 3 mm thick slices. Both mango peels and flesh slices were subsequently dried with a heat-pump dryer (WRH-100TB1S, Guangdong Weierxin Technology Co., Ltd. Foshan, China). Drying was performed at 65 °C with an air velocity of 1.8 m/s until the moisture content of the samples was reduced to less than 6%. All dried peel and flesh materials underwent preliminary coarse comminution. The coarse peel powder was incorporated into coarse mango-flesh powder at peel mass fractions of 8%, 16% and 24%. The powder blends were re-pulverized using a low-temperature vibrating ultrafine pulverizer (XDW-6B Jinan Dawei Machinery Co., Ltd. Jinan, China) to reduce the particle size (D_50_) of the powder samples to less than 30 µm. The re-pulverized pure flesh powder served as the control. All the prepared powder samples were sealed in polyester/aluminum foil/polypropylene laminated bags to avoid moisture absorption.

### 2.3. Water Adsorption Isotherms Determination and Fitting

The water adsorption isotherms of these samples were determined by the static gravimetric method [18]. Briefly, about 5 g of ultra-fine mango powder was accurately weighed and placed in a closed hermetic glass container containing different saturated salt solutions of LiCl, CH_3_COOK, MgCl_2_, K_2_CO_3_, NaBr, KI, NaCl, KCl, and BaCl_2_, whose corresponding water activity (*A_w_*) at 25 °C is 0.113, 0.225, 0.328, 0.432, 0.576, 0.689, 0.753, 0.842 and 0.901, respectively. The samples were weighed every day until the weight was less than 1 mg, at which point the equilibrium moisture was considered to have been reached. Then, the dry basis equilibrium water content *X_w_* at this point was determined using an oven method [19], and the data were fitted by the Guggenheim–Anderson–de Boer (GAB) model with Equation (1).

(1) $$X_{w}=\frac{X_{m}\cdot C\cdot K\cdot A_{w}}{\left(1-K\cdot A_{w})\cdot (1-K\cdot A_{w}+C\cdot K\cdot A_{w}\right)}$$

where *X_w_* is the dry basis equilibrium water content (g/g, dry basis, d.b.), *A_w_* is water activity, *X_m_* is the GAB monolayer water content (g/g, d.b.), and *C* and *K* are constant parameters in the mathematical model.

### 2.4. Glass Transition Temperature (T_g_) Measurement and Fitting

The glass transition temperatures of ultra-fine mango powders in equilibrium at different Aw were measured using a differential scanning calorimeter (DSC214, NETZSCH-Gerätebau GmbH, Selb, Germany). About 8 mg of each sample was placed in a hermetically sealed aluminum pan. The sample was put into the calorimeter and cooled to −90 °C at a rate of 20 °C/min, equilibrated for 5 min and then heated up to 100 °C at the same rate. Measurements were made using a sealed empty pan as a blank reference. The relationship between T_g_ and water content was fitted using the Gordon–Taylor (GT) Equation (2).

(2) $$T_{g}=\frac{X_{s}\cdot T_{\mathrm{gs}}+k\cdot X_{w}\cdot T_{\mathrm{gw}}}{X_{s}+k\cdot X_{w}}$$

where *T_g_*, *T_gs_*, and *T_gw_* are the glass transition temperatures of mango powder samples, anhydrous substrate and water (−135 °C), respectively; *X_s_* and *X_w_* are the mass fractions (g/g) of solids and water, respectively; and *k* is the constant for the interaction between the substrate and water.

### 2.5. Color Measurement

The color parameters of all powder samples were measured with a colorimeter (D25LT, Hunter Lab Co. Ltd., Reston, VA, USA). Each measurement was performed in five replicates, and the test procedure referred to the method described by Peng et al. [20]. The total color difference Δ*E* was calculated according to Equation (3), using the powder without peel addition as the reference group.

(3) $${\Delta E}^{*}=\sqrt{{\left(L^{*}-L_{0}^{*}\right)}^{2}+{\left(a^{*}-a_{0}^{*}\right)}^{2}+{\left(b^{*}-b_{0}^{*}\right)}^{2}}$$

where Δ*E* is total color difference, which denotes the color change compared to that of the control sample; L*, a*, and b* are the color parameters of the tested samples, which represent lightness, redness and yellowness; and L*_0_, a*_0_, and b*_0_ are the color parameters of the powder without peel addition.

### 2.6. Bulk Density and Angle of Repose

Both the bulk density and angle of repose are determined according to Hou et al. [21] with a few modifications. Briefly, the bulk density was measured using a 50 mL volumetric container. The container was filled with mango powder, after which the mass of the contained powder was recorded. The bulk density is the ratio of the mass of the mango powder to its volume.

The angle of repose of ultra-fine mango powder was determined via the funnel method. Briefly, 20 g of accurately weighed mango powder was loaded into a horizontal-mounted funnel for free vertical falling. The angle between the deposited powder cone and the horizontal surface was recorded with a digital angle ruler (0–200 mm, 0.8° accuracy; Wenzhou Weidu Electronics Co., Ltd., Wenzhou, China).

### 2.7. Reducing Sugar, Ascorbic Acid and β-Carotene Determination

The reducing sugar content of these ultra-fine mango powders with various peel addition was quantified using the 3,5-dinitrosalicylic acid (DNS) colorimetric method [22]. Briefly, 1.0 g of powder was mixed with 100 mL of deionized water, extracted under a 50 °C water-bath for 20 min. After filtering the supernatant, DNS reagent was added, boiled for 5 min, cooled and diluted. The absorbance was detected at 540 nm, and a glucose standard curve was used for calculation.

Quantification of ascorbic acid was performed using a high-performance liquid chromatography system equipped with an ultraviolet (UV) detector (SPD-20A, Shimadzu, Kyoto, Japan) according to Costa et al. [23].

The β-carotene was analyzed using a HPLC (LC-20AT, Shimadzu, Kyoto, Japan) equipped with a diode array detector (DAD) (model 2998; Waters, Milford, MA, USA) according to Peng et al. [24].

### 2.8. Preparation of the Extracts for Total Phenols, Total Flavonoids, and Antioxidant Activity Determination

Extraction was performed on different ultra-fine mango powder samples following Peng et al. [20], with minor modifications. Approximately 1.0 g of mango powder was accurately weighed for each sample and homogenized with 10 mL of 80% (*v*/*v*) ethanol. The mixture was subjected to ultrasonic treatment for 10 min, then centrifuged at 7155× *g* for 10 min, and the supernatant was collected. The isolation procedure was conducted two more times, and the supernatants were combined and diluted to 50 mL with 80% (*v*/*v*) ethanol.

### 2.9. Total Phenol Content (TPC) and Total Flavonoid Content (TFC) Analysis

TPC was analyzed using the Folin–Ciocalteu assay according to the Chinese national standard (GB/T 8313–2018) [25]. Specifically, 2.0 mL of the extract, 4.0 mL of deionized water, and 2.0 mL of 10% Folin reagent were mixed. After 5 min of reaction, 3.0 mL of 7.5% sodium carbonate was added and reacted under dark conditions at room temperature for 2 h. The absorbance was measured at 765 nm using a UV–vis spectrophotometer (UV-1900i, Shimadzu, Kyoto, Japan). The TPC of the mango powders was calculated and expressed in terms of gallic acid equivalents (GAE mg/g, dry basis).

TFC was analyzed using the aluminum chloride colorimetric method listed in the testing standard of China (GB/T 20574–2006) [26]. Specifically, 1.0 mL of the extract was taken, and 0.1 mL of 5% (*w*/*v*) sodium nitrite and 0.1 mL of 10% (*w*/*v*) aluminum nitrate solution were mixed sequentially. After 6 min of reaction, 1 mL of 1 mol/L sodium hydroxide was added and incubated for 15 min. The absorbance was measured at 510 nm using a UV–vis spectrophotometer. The TFC was calculated and expressed in terms of rutin equivalents (RE mg/g, dry basis).

### 2.10. Degradation Kinetics Modeling

According to Choosuk et al. [27], the nutritional quality change of the ultra-fine mango powders during storage was modeled by zero-order, first-order and second-order degradation reaction kinetics using Equations (4)–(6), respectively:

*C_t_* − *C*_0_ = −*kt* (4)

(5) $$\ln\frac{C_{t}}{C_{0}}=-\mathrm{kt}$$

(6) $$\frac{1}{C_{t}}-\frac{1}{C_{0}}=\mathrm{kt}$$

where *C_t_* is the nutrient content at day t of storage, *C*_0_ is the nutrient content at day 0, and *k* is a kinetic constant.

The half-life *t*_1/2_ of nutrient degradation can be calculated from Equation (4), Equation (5) and Equation (6), respectively, and the formulas for the zero-order, first-order and second-order are given in Equation (7), Equation (8) and Equation (9), respectively:

(7) $$t_{1/2}=\frac{C_{0}}{2k}$$

(8) $$t_{1/2}=\frac{\ln2}{k}$$

(9) $$t_{1/2}=\frac{1}{kC_{0}}$$

### 2.11. Antioxidant Activity Determination

The antioxidant capacity of the ultra-fine mango powders was determined using three complementary in vitro assays: the 2,2-diphenyl-1-picrylhydrazyl (DPPH) radical scavenging activity, 2,2-azinobis-(3-ethylbenzthiazoline-6-sulphonate (ABTS) radical scavenging activity, and ferric reducing antioxidant power (FRAP) assay, following the procedures reported by Peng et al. [20]. For each method, stand ard curves were constructed using serial dilutions of Trolox, and the results were expressed as micromoles of Trolox equivalents per gram of sample (µmol TE/g (dry basis)).

### 2.12. Statistical Analysis

All the experiments were conducted in triplicate unless specified, and all the results are presented as means ± SD. The data were analyzed using SPSS statistics software (version 21.0, SPSS Inc., Chicago, IL, USA). Statistically significant differences among the results were analyzed by one-way analysis of variance (ANOVA), followed by Duncan’s multiple comparison test at a 95% confidence level.

## 3. Results and Discussion

### 3.1. State Diagrams

**Figure 1:**
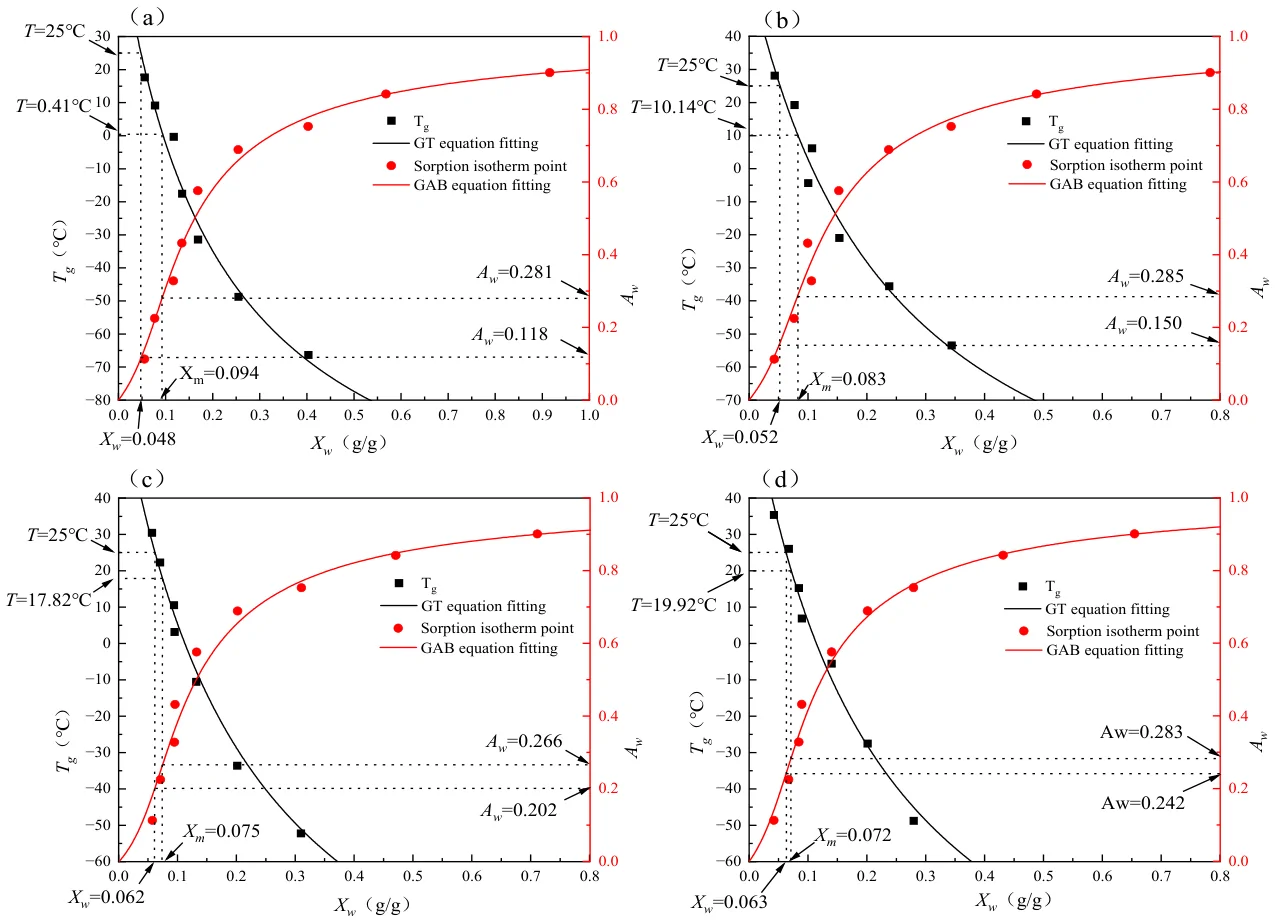
State diagrams of ultra-fine mango powders with different peel additions of 0% (**a**), 8% (**b**), 16% (**c**), and 24% (**d**).

State diagrams, derived from the combination of GAB-fitted moisture sorption isotherms and GT-fitted *T_g_* curves, provide an intuitive visualization of powder physical states across different storage environments. These diagrams are therefore indispensable for analyzing optimal storage conditions and predicting the shelf-life of fruit and vegetable powder products [28,29]. The state diagrams of ultra-fine mango powder samples are presented in Figure 1. The moisture sorption isotherms were well fitted by the GAB model with all the R^2^ values exceeded 0.990, no matter how much mango peel was added; that is to say, there was an excellent goodness-of-fit between the experimental moisture adsorption data and the GAB model predictions. Therefore, the monolayer moisture content (*X_m_*) values of the powder samples with 0%, 8%, 16%, and 24% mango peel (*w*/*w*), calculated according to the GAB model, were 0.094, 0.083, 0.075, and 0.072 g/g, respectively. The corresponding *T_g_* temperatures for these *X_m_* values were 0.41 °C, 10.14 °C, 17.82 °C, and 19.92 °C, and the associated *A_w_* values were 0.281, 0.285, 0.266, and 0.283, respectively. When the sample was at a temperature higher than its *T_g_*, the molecular mobility of the sample was increased, and the site of interaction with water molecules becomes available [29,30]. These results indicated that the incorporation of mango peel can decrease the hygroscopicity of the ultra-fine mango powders, thereby reducing their *X_m_* and increasing *T_g_*. Mango peel is rich in high-molecular-weight dietary fibers (cellulose, hemicellulose, and pectin) and polyphenols, which possess abundant hydroxyl groups. These polar functional groups can form extensive hydrogen-bonding interactions with low-molecular-weight carbohydrates (sugars and organic acids) inherently present in mango flesh powder. Such intermolecular cross-linking restricts the mobility of the carbohydrate matrix, reduces molecular chain relaxation, and increases the energy required for the matrix to transition from a glassy state to a rubbery state, leading to a higher *T_g_*. Furthermore, these peel-derived biopolymers also act as physical barriers, hindering moisture migration within the powder matrix. The elevated *T_g_* values suggest that mango peel addition can make ultra-fine mango powders maintain a glassy state at a higher moisture content level, ultimately enhancing the stability during storage.

Critical water content (CWC) is defined as the moisture content at which the *T_g_* of a sample equals ambient temperature (usually 25 °C). Below this threshold, the material remains in a glassy state, thereby exhibiting high storage stability; conversely, exceeding the CWC induces a transition to a rubbery state, which significantly compromises sample quality during preservation [30]. As shown in Figure 1, at *T_g_* = 25 °C, the CWC values of ultra-fine mango powders with 0%, 8%, 16%, and 24% mango peel (*w*/*w*), calculated according to the GT model, were 0.048, 0.052, 0.062, and 0.063 g/g, respectively. The corresponding critical *A_w_* values were 0.118, 0.150, 0.202, and 0.242, respectively. Both CWC and critical *A_w_* exhibited a significant increasing trend with increasing peel addition, indicating that mango peel effectively reduces the hygroscopicity of the ultra-fine mango powder samples. Furthermore, the *X_m_* values of all samples were consistently lower than their respective CWC values, suggesting that storage at 25 °C placed the samples in a rubbery state, thereby rendering them susceptible to quality deterioration. Hence, to better maintain the glassy state and ensure storage stability, it is advisable to either adjust the moisture content to approach the CWC or employ low-temperature storage conditions. Based on the above state diagram analysis, all ultra-fine mango powder samples sealed in polyester/aluminum foil/polypropylene laminated bags were subsequently stored at 4 °C, and their quality changes were evaluated under this storage condition.

### 3.2. Color Variation of Ultra-Fine Mango Powder During Storage

**Figure 2:**
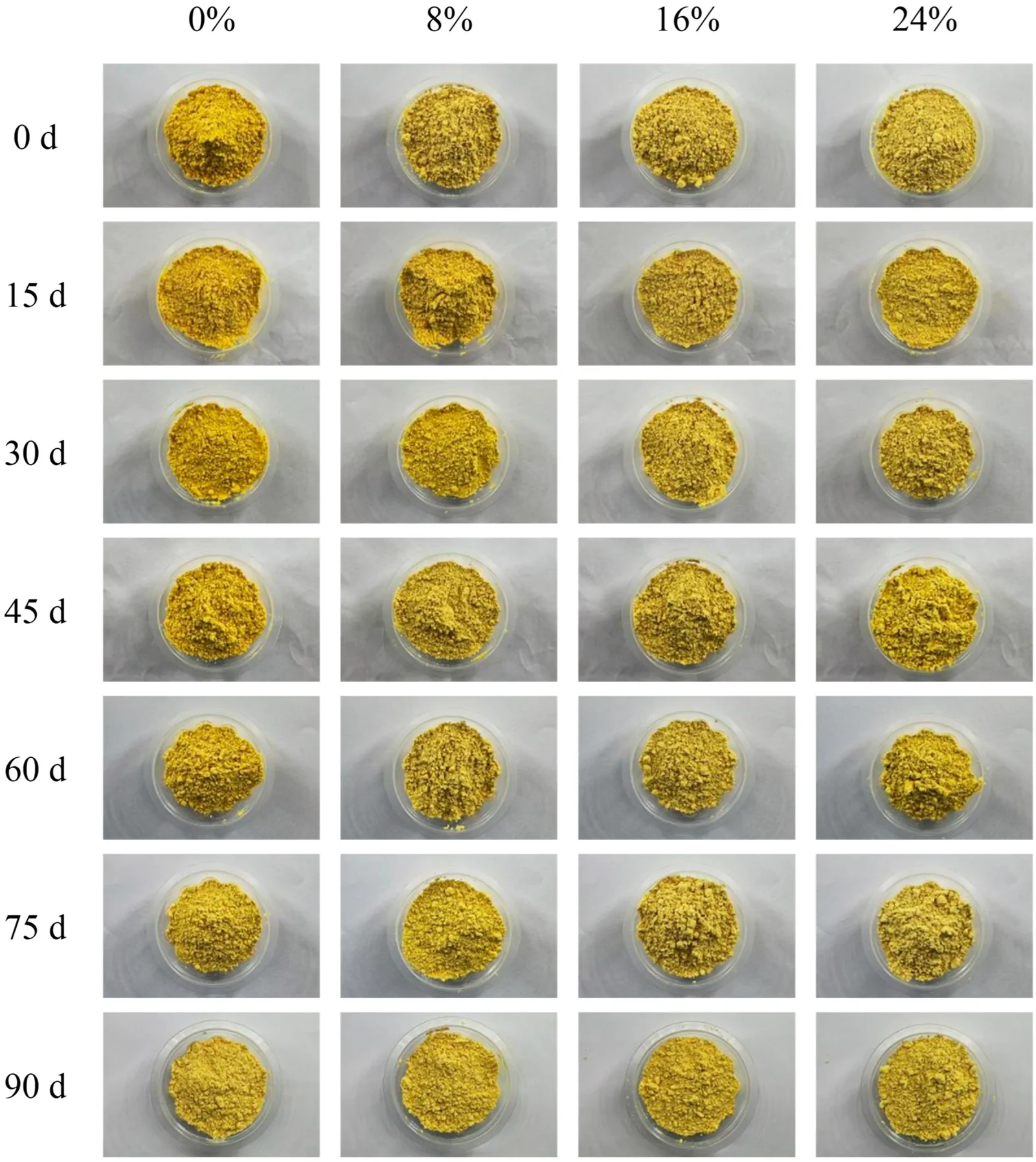
Changes in appearance of ultra-fine mango powders with different peel addition during storage from 0 to 90 days.

Color is a primary quality attribute of mango powder that significantly influences consumers’ acceptance and purchasing behavior. As shown in Table 1, the incorporation of peel into ultra-fine mango powder resulted in a progressive increase in L* values, whereas both a* and b* values exhibited an overall decreasing trend with increasing peel addition. That is, the addition of the peel increased the brightness, but weakens the redness and yellowness of the samples (Figure 2). During subsequent storage, L* values of all samples showed a continuous increase, while the a* values showed a consistent decline. Consequently, all the ultra-fine mango powders exhibited a progressive increase in lightness and a reduction in yellowness over the storage period. A similar variation trend was also observed in Refractance Window^®^-dried mango powder stored at 5 °C [31]. Different from the variation trend of L* values and a* values, the b* values of all samples increased after 15 days of storage, followed by a gradual decrease from 30 to 90 days. The observed color alterations during storage are likely attributable to multiple concomitant reactions. The degradation of intrinsic chromophoric compounds primarily accounted for the overall color shift. Specifically, the increase in L* and the decrease in a* values in our study were mainly ascribed to the degradation of β-carotene, given carotenoids are susceptible to such reactions under low-temperature (4 °C) storage conditions [32]. It is undeniable that the degradation of β-carotene also results in a loss of nutritional value of the ultra-fine mango powder.

According to the Δ*E* values (Table 1), the highest total color difference (Δ*E* = 10.73) was obtained in the sample without peel addition after 90 days of storage, and a visible color difference can also be observed (Figure 2). Notably, the Δ*E* values of the samples with peel incorporation were significantly lower than the pure mango powder throughout the entire storage period. After 90 days of storage, the Δ*E* values for samples containing 8%, 16%, and 24% (m/m) mango peel were 2.82, 3.51, and 1.85, respectively. These findings suggest that the incorporation of mango peel effectively mitigates color deterioration, potentially by inhibiting the Maillard reaction, enzymatic browning, and carotenoids degradation. This protective effect is likely attributable to the abundant polyphenols present in mango peel, which are known for their potent antioxidant capacity [33]. Our results are consistent with those of Tan et al. [3], who reported that green tea polyphenols (GTPs) could inhibit carotenoid degradation via free radical scavenging, thereby stabilizing the color attributes of mango powder.

### 3.3. Moisture Content and Aw Variation of Ultra-Fine Mango Powder During Storage

**Figure 3:**
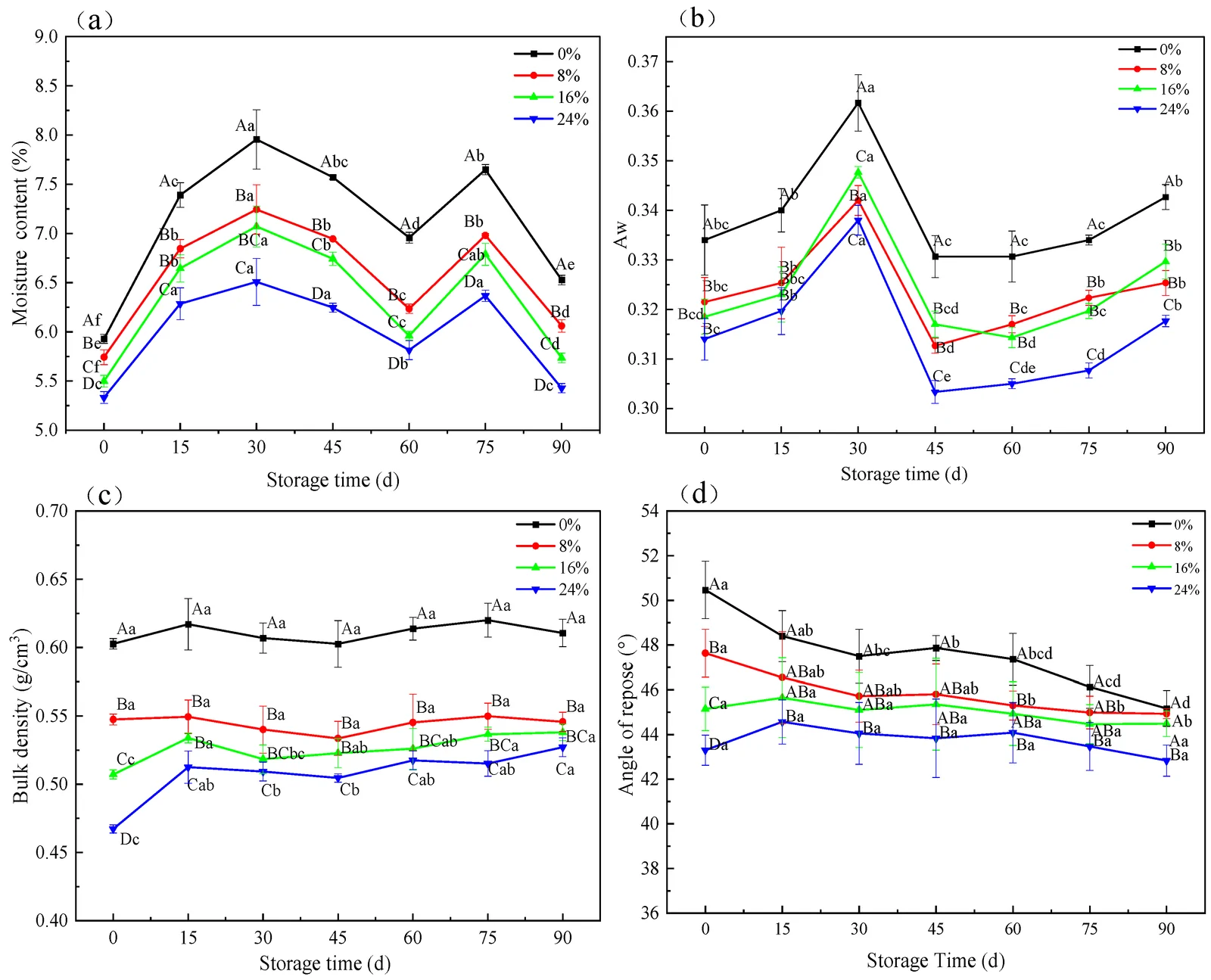
Change in moisture content (**a**), water activity (*A_w_*) (**b**), bulk density (**c**), and angle of repose (**d**) of ultra-fine mango powders with different peel addition during storage. Different upper-case letters in the figure represent significant differences (*p* < 0.05) among different peel additions of ultra-fine mango powder under the same storage time; different lower-case letters represent significant differences among different storage times under the same peel addition (*p* < 0.05).

The moisture content of all samples increased sharply during the initial 30 days of storage (Figure 3a), which may be attributed to the strong hygroscopicity of reducing sugars present in mango powder. The polyester/aluminum foil/polypropylene laminated bag used for mango powder packaging provides an excellent barrier against water vapor and oxygen; hence these reducing sugars only absorbed the moisture within the packaging environment [34]. Beyond 30 days, the moisture contents of the samples exhibited an approximately downward oscillating trend. Conspicuously, the incorporation of peel effectively inhibited moisture adsorption in the ultra-fine mango powders, and this inhibitory effect intensified with increasing peel addition levels.

The Aw of all samples followed a trend similar to that of moisture content during the first 45 days; thereafter, Aw gradually increased with storage time (Figure 3b), which was different from the variation trend of moisture content. This discrepancy may be explained by two factors: on one hand, the sugars in mango powder adsorbed moisture and subsequently underwent crystallization, which released previously bound water [35]; on the other hand, prolonged storage may have altered the structural properties of mango powder, such as particle arrangement and porosity, thereby influencing its moisture release capacity [36]. Importantly, peel addition significantly reduced the Aw as well, enabling simultaneous maintenance of low moisture content and Aw, thus contributing to improved storage stability of ultra-fine mango powder.

### 3.4. Physical Properties Variation of Ultra-Fine Mango Powder During Storage

Bulk density and repose angle are widely employed to quantify powder flowability, with lower values generally indicating reduced particle agglomeration and superior flow properties. As depicted in Figure 3c, no significant difference in bulk density was observed for samples containing 0% and 8% mango peel during the 90-day storage period. In contrast, samples with 16% and 24% peel addition exhibited a slight increase within the initial 15 days, which may be attributed to enhanced van der Waals forces among the powder particles as they stay closer in the packed environment, making them more compact [37]. In addition, dietary fibers originating from mango peel act as physical barriers and thereby exert an anti-caking effect. Nevertheless, throughout the entire storage duration, all peel-enriched formulations maintained consistently lower bulk density values relative to the control. Regarding the angle of repose, both the control and the low-peel-addition (8%) samples exhibited a gradual decline over the storage period, whereas no significant changes were detected in the 16% and 24% peel addition samples (Figure 3d). Collectively, these findings suggested that addition of mango peel not only mitigated moisture adsorption but also modified the powder structural matrix, thereby simultaneously reducing the bulk density and angle of repose, and ultimately enhancing powder flowability.

### 3.5. Phytochemical Compound Variation of Ultra-Fine Mango Powder During Storage

**Figure 4:**
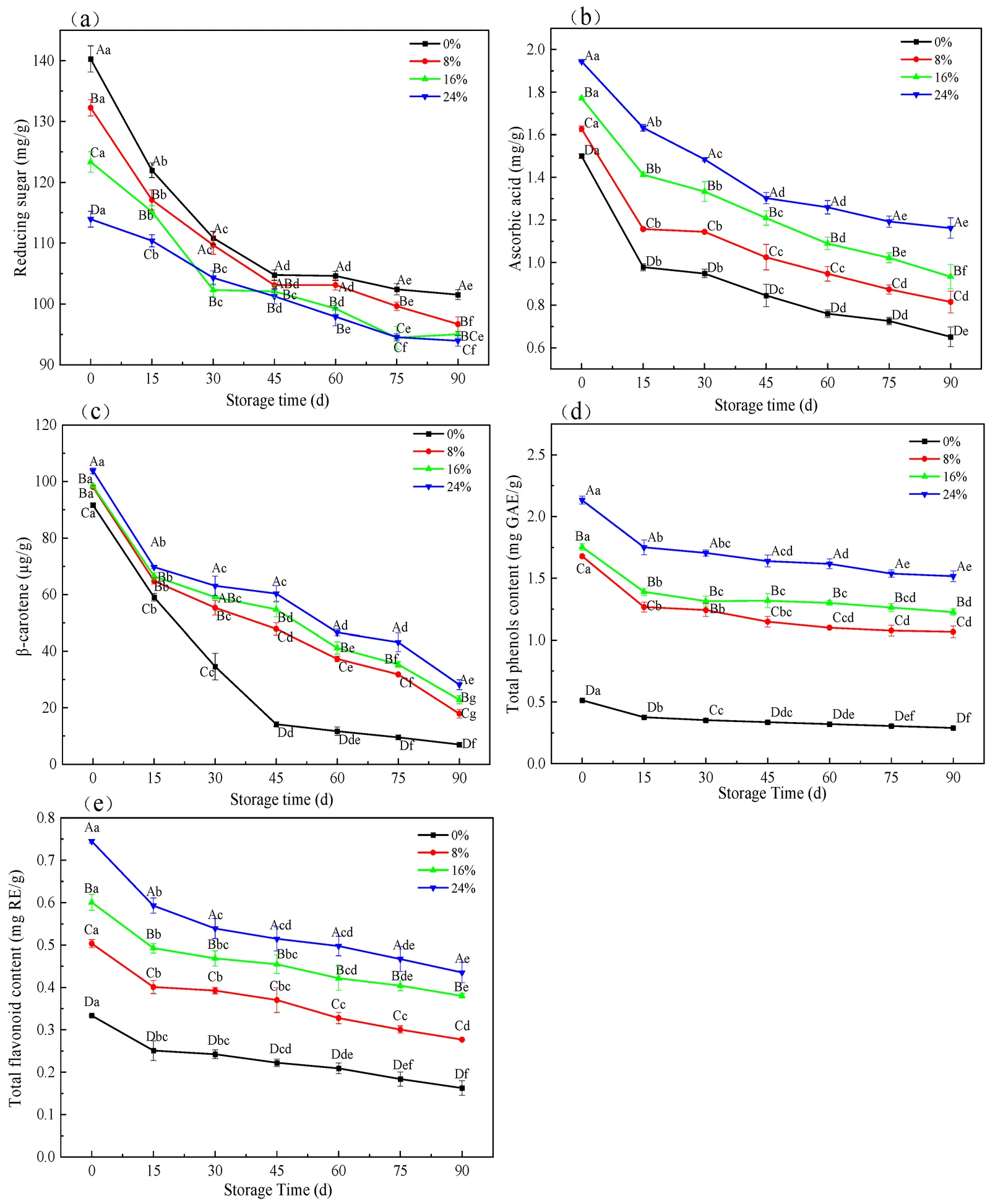
The nutriments of reducing sugar (**a**), ascorbic acid (**b**), β-carotene (**c**), total phenol (**d**), and total flavonoid (**e**) variation of ultra-fine mango powders with different peel addition during storage. Different upper-case letters in the figure represent significant differences (*p* < 0.05) among different peel additions of ultra-fine mango powder under the same storage time; different lower-case letters represent significant differences among different storage times under the same peel addition (*p* < 0.05).

The changes in reducing sugar, ascorbic acid, and β-carotene content of the ultra-fine mango powder samples during 90 days of storage at 4 °C are presented in Figure 4a–c. A general declining trend in reducing sugar, ascorbic acid, and β-carotene content was observed across all samples. After 90 days of storage, mango powders supplemented with 0%, 8%, 16%, and 24% mango peel exhibited reduction ratios of 27.62%, 26.91%, 22.96%, and 17.53% for reducing sugar; 56.67%, 49.99%, 47.46%, and 40.20% for ascorbic acid; and 92.37%, 81.72%, 76.75%, and 72.84% for β-carotene, respectively. The reduction in detectable reducing sugars during storage is mainly ascribed to non-browning complexation with dietary fiber and polyphenols from mango peel via hydrogen bonding [38], instead of the Maillard reaction, which aligns with the absence of visible browning of these samples (Figure 2). Meanwhile, the reduction in ascorbic acid and β-carotene was highly susceptible to oxidative degradation. In any case, these results confirm that mango peel incorporation significantly inhibited the reduction in reducing sugar, ascorbic acid, and β-carotene, and with higher peel content yielding stronger stabilization effects. Notably, the reducing rates of all these three indicators were quicker in the early storage period (0–45 days) than the later storage period (45–90 days). This accelerated early-stage degradation is mainly due to the fact that mango powder adsorbed moisture from the environment during the initial storage (Figure 3a). The resultant water activity of the samples was sufficiently high to trigger intensified degradation reactions of these phytochemical compounds [39]. In agreement with these observations, González et al. [40] also reported enhanced degradation of lycopene and ascorbic acid in grapefruit powder stored under high relative humidity conditions.

The changes in TPC and TFC of ultra-fine mango powders with various mango peel addition during storage are illustrated in Figure 4d,e. The incorporation of mango peel substantially increased the baseline levels of TPC and TFC. Specifically, TPC rose from 0.51 mg GAE/g (0% peel) to 1.68, 1.75, and 2.13 mg GAE/g for the 8%, 16%, and 24% peel-supplemented samples, respectively. Correspondingly, TFC increased from 0.33 mg RE/g (0% peel) to 0.50, 0.60, and 0.74 mg RE/g, respectively. This is mainly due to the fact that mango peel possesses significantly higher content of phenolic compounds and flavonoids compared to the peels of other fruits [41]. Notably, the observed increments in both TPC and TFC were disproportionately greater than the percentages of peel added. Under the premise that all samples were subjected to the same extraction conditions, this discrepancy likely results from the introduction of peel-derived bioactive constituents rich in phenolic hydroxyl groups, which are presumed to interact synergistically with the endogenous phenolic compounds in the pulp matrix, potentially enhancing the overall chromogenic reactivity in the assays [13,42], thereby yielding a greater than expected increase in the determined TPC and TFC. Generally, similar synergistic effects have also been observed during the processing of apple peel powder [43]. During storage, a declining trend for TPC and TFC was also observed in all samples, similar to the trend observed for ascorbic acid. After 90 days of storage, mango powders containing 0%, 8%, 16%, and 24% mango peel exhibited reduction ratios of 43.60%, 36.44%, 30.03%, and 28.91% for TPC, and 51.24%, 44.96%, 36.70%, and 41.58% for TFC, respectively. These results further confirm that the incorporation of mango peel mitigated the degradation of TPC and TFC throughout the storage period, with the protective effect being generally enhanced at higher peel incorporation levels.

### 3.6. Degradation Kinetics of Phytochemical Compounds

The mathematical kinetic model is an extremely useful tool for food quality prediction during storage. Table 2 shows the results of a regression analysis of the zero-order, first-order and second-order reaction kinetics in ultra-fine mango powders with various mango peel addition during storage. According to correlation coefficients (R^2^), the degradation processes of reducing sugar and β-carotene in these samples were best described by the first-order kinetic equation, with R^2^ values of 0.8727–0.9725 and 0.9368–0.9573, while ascorbic acid, TPC, and TFC in these samples were best described by the second-order kinetic equation, with R^2^ values of 0.9402–0.9874, 0.7180–0.8835, and 0.9377–0.9716, respectively. Similarly, several authors have reported in the literature that first-order or second-order kinetic models can well describe the degradation of phytochemical compounds in coconut chips [27] and mango powder during storage [2].

Kinetic parameters (*k*) of reducing sugar, ascorbic acid, β-carotene, TPC, and TFC fitted with their respective optimal kinetic models are summarized in Table 3. Notably, the estimated *k* values for ascorbic acid decreased progressively with increasing mango peel addition, from 0.0085 (0% peel) to 0.0062 (8%), 0.0053 (16%), and 0.0039 (24%); the corresponding half-lives (*t*_1/2_) of ascorbic acid were 78.43, 99.10, 106.54, and 131.88 days, respectively. This trend indicates that the incorporation of mango peel effectively retarded the degradation of ascorbic acid during storage. Similarly, the degradation rates of reducing sugars, β-carotene, TPC, and TFC were also attenuated in a peel-addition-dependent manner. Collectively, these kinetic data provide a rational basis for shelf-life prediction of ultra-fine mango powders with varying peel contents, based on the stability of their bioactive phytochemicals.

### 3.7. Antioxidant Activity Variation of Ultra-Fine Mango Powder During Storage

**Figure 5:**
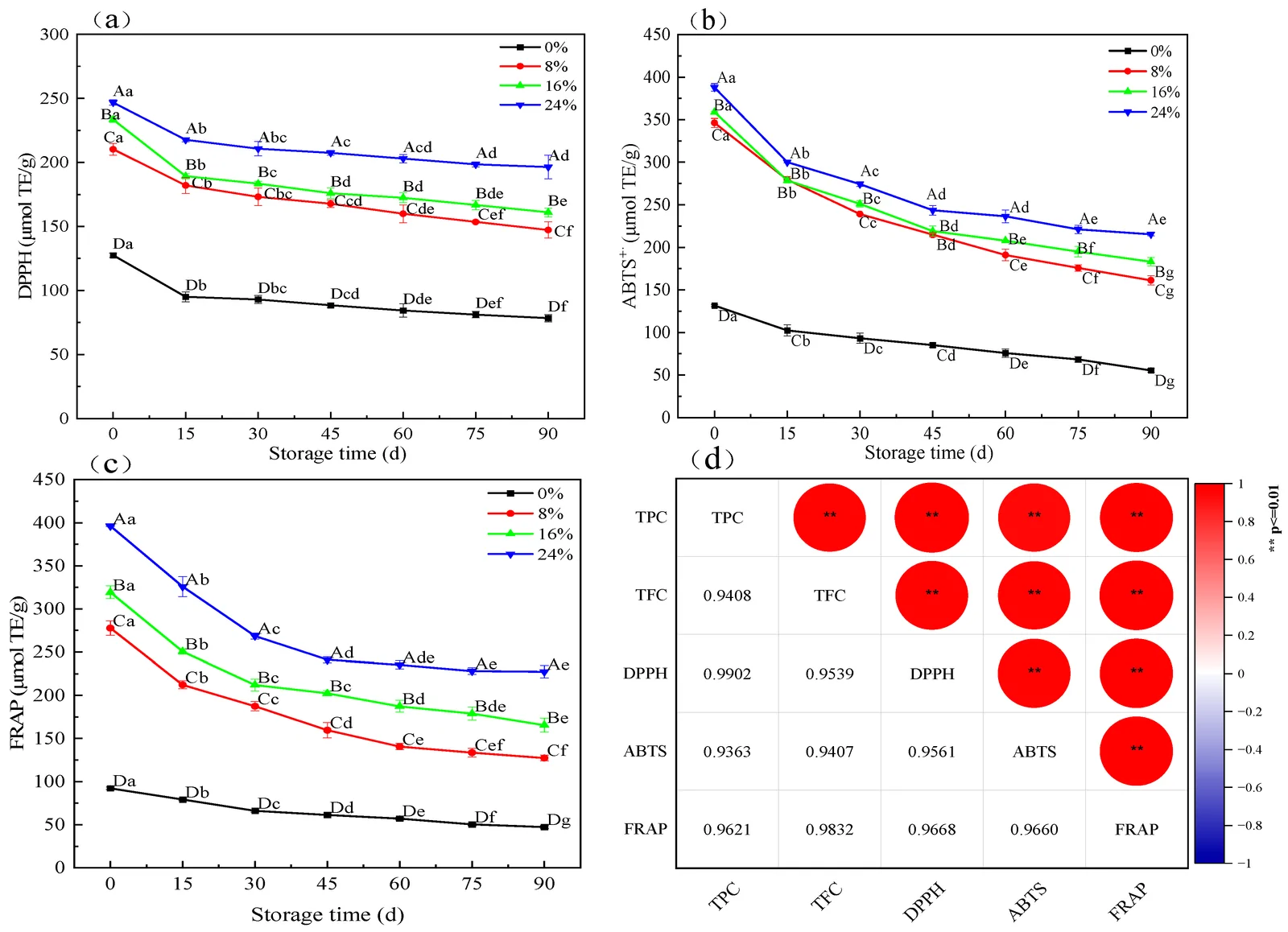
The antioxidant activity of DPPH (**a**), ABTS (**b**), and FRAP (**c**) variations and their correlation with total phenols and total flavonoids (**d**) in ultra-fine mango powders with different peel addition during storage. Different upper-case letters in the figure represent significant differences (*p* < 0.05) among different peel additions of ultra-fine mango powder under the same storage time; different lower-case letters represent significant differences among different storage times under the same peel addition (*p* < 0.05).

The changes in the antioxidant activity of ultra-fine mango powders during storage are presented in Figure 5a–c. For samples with the same peel addition and storage time, obvious variations in antioxidant activity were observed depending on the analytical methods employed. Such discrepancies may be dependent on the existence of multiple radical species, the diverse physical and chemical characteristics of the oxidants involved, and corresponding differences in reaction mechanisms [44]. On the whole, the antioxidant activity of all ultra-fine mango powders showed a significant decrease during storage regardless of which analytical method was used, and peel addition could slow down this downward trend. Specifically, the DPPH free radical scavenging activity of mango powders supplemented with 0%, 8%, 16%, and 24% mango peel decreased from 127.53, 210.24, 233.39, and 246.78 μmol TE/g to 78.39, 147.20, 160.94, and 196.37 μmol TE/g after 90 days of storage, corresponding to reduction ratios of 38.53%, 29.99%, 31.04%, and 20.43%, respectively. The degradation trend of antioxidant activity was highly consistent with TPC and TFC, and the correlation values (R^2^) among antioxidant activity, TPC, and TFC were all over 0.9407, indicating an extremely significant correlation (*p* ≤ 0.01) among these indicators (Figure 5d). This is attributable to the fact that phenolic compounds and flavonoids constitute the primary source of antioxidant capacity in fruit powder products. These findings are consistent with previous studies on apple powder [43], blueberry powder [45], and chokeberry pulp powder [46], all of which reported a significant positive correlation between antioxidant activity and the contents of phenolic compounds and flavonoids.

## 4. Conclusions

This research demonstrates incorporating mango peel (0–24%, *w*/*w*) into ultra-fine mango powder significantly enhanced storage stability, as evidenced by state diagram analysis showing elevated *T_g_* and critical water content. Although quality deterioration of all ultra-fine mango powders occurred during 90 days of storage at 4 °C, peel addition consistently mitigated these physicochemical changes throughout the whole storage period. The L* values of powder samples increased and a* values decreased during storage due to the oxidative and photodegradative degradation of color phytochemical compounds, but the peel addition narrowed the color change. The incorporation of peel effectively inhibited moisture adsorption and A_w_ in the ultra-fine mango powders, thereby ensuring the microbiological safety of the powders. Also, it enhanced the powder flowability during storage, since mango peel contains high dietary fiber content. Notably, the degradation of reducing sugars and β-carotene followed first-order kinetics, whereas ascorbic acid, TPC, and TFC conformed to second-order models; peel incorporation not only improved the initial levels of the above phytochemical compounds but also reduced their degradation rate constants and extended half-lives (T_1/2_) in a dose-dependent manner. Furthermore, the antioxidant activity of ultra-fine mango powders, which exhibited a strong positive correlation with TPC and TFC (R^2^ > 0.94), was significantly better preserved in peel-enriched formulations, corroborating the protective role of phenolic compounds against oxidative deterioration. Collectively, the results provide valuable insights into the mechanisms and rates of changes in color, flowability, and bioactive components, and the models describing the storage process of ultra-fine mango powder also offer a sustainable strategy for by-product utilization for improving the storage stability of fruit powder samples. Future research should address sensory attributes and underlying synergistic mechanisms between peel polyphenols and pulp matrix components.

## Figures and Tables

**Table 1:** Changes in color parameters of the ultra-fine mango powders with different peel addition during storage.

	Time/d	Peel Content/%			
		0	8	16	24
L*	0	79.22 ± 0.86 ^Cf^	81.86 ± 0.49 ^Bd^	82.09 ± 0.56 ^Bd^	83.25 ± 0.32 ^Ad^
	15	81.07 ± 0.08 ^Ce^	82.91 ± 0.09 ^Bc^	82.71 ± 0.10 ^Bc^	83.32 ± 0.08 ^Ad^
	30	81.65 ± 0.17 ^Cd^	82.91 ± 0.31 ^Bc^	83.46 ± 0.16 ^Ab^	83.56 ± 0.24 ^Ad^
	45	83.10 ± 0.05 ^Cc^	84.16 ± 0.10 ^Ba^	84.20 ± 0.11 ^Ba^	84.78 ± 0.12 ^Aa^
	60	83.11 ± 0.20 ^Dc^	83.58 ± 0.07 ^Cb^	83.98 ± 0.27 ^Ba^	84.63 ± 0.37 ^Aab^
	75	83.62 ± 0.12 ^Bb^	83.77 ± 0.09 ^Bb^	83.92 ± 0.06 ^ABa^	84.14 ± 0.34 ^Ac^
	90	84.09 ± 0.21 ^Aa^	84.09 ± 0.13 ^Aa^	84.14 ± 0.17 ^Aa^	84.31 ± 0.18 ^Abc^
a*	0	6.30 ± 0.06 ^Aa^	3.99 ± 0.13 ^Ba^	4.04 ± 0.06 ^Ba^	3.58 ± 0.12 ^Ca^
	15	5.88 ± 0.04 ^Ab^	4.01 ± 0.05 ^Ba^	4.06 ± 0.03 ^Ba^	3.44 ± 0.03 ^Cb^
	30	5.22 ± 0.05 ^Ac^	3.81 ± 0.12 ^Bb^	3.54 ± 0.08 ^Cd^	3.14 ± 0.06 ^Dd^
	45	4.54 ± 0.05 ^Ad^	3.79 ± 0.03 ^Bbc^	3.71 ± 0.02 ^Bb^	3.12 ± 0.03 ^Ce^
	60	4.11 ± 0.04 ^Ae^	3.64 ± 0.02 ^Bd^	3.57 ± 0.04 ^Bd^	3.06 ± 0.03 ^Cd^
	75	3.75 ± 0.05 ^Af^	3.70 ± 0.05 ^Acd^	3.70 ± 0.03 ^Abc^	3.25 ± 0.09 ^Bd^
	90	3.70 ± 0.03 ^Af^	3.68 ± 0.06 ^Ad^	3.64 ± 0.07 ^Ac^	3.34 ± 0.05 ^Bc^
b*	0	41.29 ± 0.41 ^Ab^	35.70 ± 0.39 ^Bb^	35.72 ± 0.32 ^Bb^	32.99 ± 0.12 ^Cb^
	15	42.06 ± 0.09 ^Aa^	37.27 ± 0.07 ^Ba^	37.45 ± 0.07 ^Ba^	34.48 ± 0.08 ^Ca^
	30	38.52 ± 0.16 ^Ac^	35.44 ± 0.23 ^Bb^	34.23 ± 0.17 ^Cc^	32.97 ± 0.20 ^Db^
	45	35.17 ± 0.31 ^Ad^	34.30 ± 0.30 ^Bc^	33.62 ± 0.32 ^Cd^	31.61 ± 0.14 ^Dc^
	60	33.66 ± 0.14 ^Be^	34.26 ± 0.31 ^Ac^	33.07 ± 0.33 ^Ce^	31.04 ± 0.27 ^Dd^
	75	32.40 ± 0.16 ^Cf^	34.29 ± 0.29 ^Ac^	33.18 ± 0.17 ^Be^	31.58 ± 0.21 ^Dc^
	90	32.09 ± 0.25 ^Cg^	34.01 ± 0.08 ^Ac^	32.91 ± 0.09 ^Be^	31.51 ± 0.25 ^Dc^
ΔE*	0	-	-	-	-
	15	2.05 ± 0.07 ^Af^	1.90 ± 0.07 ^Bd^	1.84 ± 0.07 ^Bd^	1.50 ± 0.08 ^Cc^
	30	3.85 ± 0.11 ^Ae^	1.25 ± 0.18 ^Ce^	2.17 ± 0.10 ^Bc^	0.59 ± 0.16 ^Dd^
	45	7.46 ± 0.26 ^Ad^	2.71 ± 0.18 ^Cc^	3.07 ± 0.07 ^Bb^	2.11 ± 0.16 ^Dab^
	60	8.85 ± 0.04 ^Ac^	2.43 ± 0.16 ^Cb^	3.31 ± 0.19 ^Bab^	2.47 ± 0.23 ^Ca^
	75	10.24 ± 0.14 ^Ab^	2.48 ± 0.18 ^Cb^	3.14 ± 0.17 ^Bb^	1.71 ± 0.35 ^Dbc^
	90	10.73 ± 0.22 ^Aa^	2.82 ± 0.09 ^Ca^	3.51 ± 0.13 ^Ba^	1.85 ± 0.21 ^Db^

Note: Different upper-case letters in the table represent significant differences among different peel additions of ultra-fine mango powder with the same storage time, (*p* < 0.05); different lower-case letters represent significant differences among different storage times under the same peel addition (*p* < 0.05).

**Table 2:** Kinetic model fitting coefficients of the total phenols, total flavonoids, ascorbic acid, β-carotene and reducing sugar of the ultra-fine mango powders with different peel addition during storage.

	Peel Content/%	Determination Coefficients of Zero-Order *R*^2^	Determination Coefficients of First-Order *R*^2^	Determination Coefficients of Second-Order *R*^2^
Reducing sugar	0	0.7728	0.9280	0.8257
	8	0.8629	0.8891	0.9124
	16	0.8557	0.8727	0.8883
	24	0.9655	0.9725	0.9782
Ascorbic acid	0	0.7785	0.8720	0.9402
	8	0.8257	0.8978	0.9494
	16	0.9228	0.9655	0.9874
	24	0.8835	0.9214	0.9516
β-Carotene	0	0.8200	0.9520	0.9634
	8	0.9209	0.9549	0.8220
	16	0.9246	0.9573	0.8688
	24	0.8980	0.9368	0.8676
TPC	0	0.7530	0.8216	0.8835
	8	0.7190	0.7710	0.8215
	16	0.6430	0.6809	0.7180
	24	0.7556	0.8062	0.8445
TFC	0	0.8951	0.9436	0.9570
	8	0.9252	0.9578	0.9716
	16	0.877	0.9207	0.9503
	24	0.8406	0.8946	0.9377

**Table 3:** Kinetic model parameters of the total phenols, total flavonoids, ascorbic acid, β-carotene, and reducing sugar of the ultra-fine mango powders with different peel addition during storage.

	Peel Content/%	Prediction Functions	*k*	*t*_1/2_/d
Reducing sugar	0	*C_t_* = 140.2695e^−0.0032*t*^	0.0032	214.60
	8	*C_t_* = 132.2496e^−0.0032*t*^	0.0032	217.29
	16	*C_t_* = 123.3343e^−0.0029*t*^	0.0029	239.02
	24	*C_t_* = 113.9289e^−0.0023*t*^	0.0023	304.01
Ascorbic acid	0	*C_t_* = 1/(0.0085*t* + 0.6666)	0.0085	78.43
	8	*C_t_* = 1/(0.0062*t* + 0.6144)	0.0062	99.10
	16	*C_t_* = 1/(0.0053*t* + 0.5647)	0.0053	106.54
	24	*C_t_* = 1/(0.0039*t* + 0.5143)	0.0039	131.88
β-Carotene	0	*C_t_* = 91.6581e^−0.0296*t*^	0.0296	23.42
	8	*C_t_* = 98.0738e^−0.0165*t*^	0.0165	42.01
	16	*C_t_* = 98.3863e^−0.0143*t*^	0.0143	48.47
	24	*C_t_* = 103.8256e^−0.0123*t*^	0.0123	56.35
TPC	0	*C_t_* = 1/(0.0144*t* + 1.9454)	0.0144	135.10
	8	*C_t_* = 1/(0.0034*t* + 0.5959)	0.0034	175.28
	16	*C_t_* = 1/(0.0021*t* + 0.5705)	0.0021	271.65
	24	*C_t_* = 1/(0.0018*t* + 0.4688)	0.0018	260.47
TFC	0	*C_t_* = 1/(0.0314*t* + 2.9981)	0.0314	95.48
	8	*C_t_* = 1/(0.0168*t* + 1.9874)	0.0168	118.30
	16	*C_t_* = 1/(0.0196*t* + 1.6649)	0.0196	173.43
	24	*C_t_* = 1/(0.0094*t* + 1.3428)	0.0094	142.85

## Data Availability

The datasets generated and/or analyzed during the current study are available from the first author on reasonable request.
